# A role of the claustrum in auditory scene analysis by reflecting sensory change

**DOI:** 10.3389/fnsys.2014.00044

**Published:** 2014-04-04

**Authors:** Ryan Remedios, Nikos K. Logothetis, Christoph Kayser

**Affiliations:** Max Planck Institute for Biological CyberneticsTübingen, Germany; Division of Biology,California Institute of TechnologyPasadena, CA, USA; Division of Imaging Science and Biomedical Engineering, University of ManchesterManchester, UK; Institute of Neuroscience and Psychology, University of GlasgowGlasgow, UK

**Keywords:** claustrum, saliency, auditory response, sounds, vocalizations, insula

## Abstract

The biological function of the claustrum remains speculative, despite many years of research. On the basis of its widespread connections it is often hypothesized that the claustrum may have an integrative function mainly reflecting objects rather than the details of sensory stimuli. Given the absence of a clear demonstration of any sensory integration in claustral neurons, however, we propose an alternative, data-driven, hypothesis: namely that the claustrum detects the occurrence of novel or salient sensory events. The detection of new events is critical for behavior and survival, as suddenly appearing objects may require rapid and coordinated reactions. Sounds are of particular relevance in this regard, and our conclusions are based on the analysis of neurons in the auditory zone of the primate claustrum. Specifically, we studied the responses to natural sounds, their preference to various sound categories, and to changes in the auditory scene. In a test for sound-category preference claustral neurons responded to but displayed a clear lack of selectivity between monkey vocalizations, other animal vocalizations or environmental sounds (Esnd). Claustral neurons were however able to detect target sounds embedded in a noisy background and their responses scaled with target signal to noise ratio (SNR). The single trial responses of individual neurons suggest that these neurons detected and reflected the occurrence of a change in the auditory scene. Given its widespread connectivity with sensory, motor and limbic structures the claustrum could play the essential role of identifying the occurrence of important sensory changes and notifying other brain areas—hence contributing to sensory awareness.

## Introduction

The biological function of the claustrum as a brain structure remains speculative, despite many years of research. Past and present hypotheses (Edelstein and Denaro, 2004; Crick and Koch, 2005; Smythies et al., 2012) proposed a function based on the claustrum’s most pronounced feature: its widespread anatomical connections throughout the brain. The claustrum reciprocally and topographically connects cortical areas and subcortical structures including both early sensory and higher association regions (Pearson et al., 1982; Sadowski et al., 1997; Tanné-Gariépy et al., 2002; Fernandez-Miranda et al., 2008; Park et al., 2012; Milardi et al., 2013). This connectivity suggests an integrative function utilizing the afferents from multiple brain regions. This notion of an integrative function was supported by findings of single neuron studies reporting that claustral neurons respond to sensory stimulation in visual, acoustic, and somatic modalities, hence based on experimental evidence for a convergence of afferent multisensory information (Segundo and Machne, 1956; Spector et al., 1970, 1974; Olson and Graybiel, 1980; Clarey and Irvine, 1986; Sherk, 1986). Similar conclusions were also drawn based on results in human functional imaging studies (Hadjikhani and Roland, 1998; Banati et al., 2000). Based on the overall anatomical and functional evidence, and by drawing analogies between claustrum and other integrative brain structures, it has been proposed that the claustrum serves as an integrator of sensory information (Edelstein and Denaro, 2004; Crick and Koch, 2005; Smythies et al., 2012).

Yet, alternatively, the strong connectivity of claustrum may in principle reflect its capacity to impact on multiple brain structures. Indeed, direct attempts to uncover claustral neural functions typically associated with multisensory processing or integration have failed. For example, we have previously studied claustral neurons during audio-visual stimulation (Remedios et al., 2010). Using established indices of non-trivial multisensory neural-response properties we systematically probed claustral neurons for typical signs of response enhancement or depression known from other multisensory structures (Kayser and Logothetis, 2007; Stein and Stanford, 2008). Surprisingly, we found that the vast majority of neurons responded only to visual or auditory stimuli and very few neurons exhibited statistically significant multisensory response interactions. This data, on one hand, confirm the multisensory nature of the claustrum as a structure consisting of distinct and separated unisensory zones, and on the other hand refute the idea of claustrum being a direct sensory integrator; a notion also supported by more recent work (Smith et al., 2012). While these results do not necessarily rule out a role in handling or routing information from multiple sensory modalities in general (Smythies et al., 2012), they suggest that the primary function of the claustrum is a different one than the direct integration and merging of multisensory information.

Based on data obtained in a previous study we have suggested that the claustrum may serve as a detector for the occurrence of novel or salient sensory events—saliency here referring to an acoustic difference between a specific sound token and the background auditory scene (Remedios et al., 2010). This hypothesis is not only consistent with our observation of relatively transient responses to auditory stimuli in the primate claustrum (Remedios et al., 2010) but also fits well with reported properties of visual neurons in the claustrum. Early pioneering studies on the visual claustrum in the cat found an overrepresentation of the peripheral visual field, that claustral neurons are broadly tuned and respond best to large and moving, hence salient, visual stimuli (Olson and Graybiel, 1980; Sherk and Levay, 1981a). In addition, the claustrum has anatomical connections with parietal and frontal areas involved in saliency processing (Bogler et al., 2011). In a function as novelty or saliency detector the claustrum would be more involved in reporting the appearance of a new sensory events rather than encoding their specific configural attributes. It subsequently would report the occurrence of such events to a wide-spread network of cortical and subcortical areas to trigger additional sensory processing or guide immediate behavioral reactions.

The detection of novel sensory events is critical for behavior and survival, as suddenly appearing objects may require rapid and coordinated reactions. Sounds are of particular relevance in this regard, as they can carry warning signals that can be perceived regardless of state of vigilance or direction of gaze (Issa and Wang, 2008). We here elaborate on the still speculative hypothesis about a role of the claustrum in novelty detection based on experimental data that makes a step towards testing this hypothesis more directly. Specifically, we consider auditory responsive neurons in the auditory zone of the claustrum and study their response properties with regard to encoding sound categories and detecting novel sounds. Overall our data provide evidence that is more consistent with a role in sound onset detection than a role in encoding information about the detailed nature of the respective sensory event.

## Materials and methods

**Figure 1:**
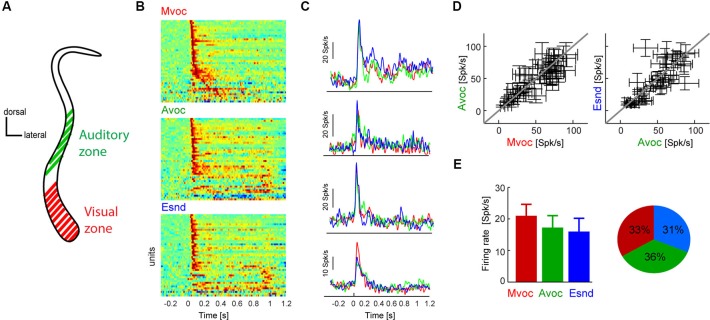
**(A)** Schematic coronal section through the claustrum showing the putative location of the auditory and visual sensory zones. In previous work we have established a visually dominant and an auditory dominant zone along the dorso-ventral extend of the claustrum, the approximate locations of which are indicated in the figure. Please refer to previous work for an in-depth description of the anatomical localization of these regions (Remedios et al., 2010). The neurons analyzed here were all recorded in the auditory zone. **(B)** Diagram showing each units average response to each sound category. For display purpose each time course was scaled to within the same range, and red colors indicate periods of high response amplitude (*n* = 45 units). Sounds start at *t* = 0 and lasted between 0.3 and 1 s. **(C)** Example data from four units, showing the average response time course within each category. Except for the bottom example, responses were highly similar between sound categories. **(D)** Response amplitudes for all units and sound categories. Responses were calculated in 100 ms windows centered on the peak response for each individual sound. Errorbars indicate each units’ average responses to each category and the standard error across sounds within each category. **(E)** Population averaged response amplitudes (mean and s.e.m.) and the fraction of units “preferring” each category (defined based on the maximal response amplitude). Sound categories: Mvoc: conspecific vocalizations; Avoc: sounds of other animals; Esnd: environmental sounds).

Two adult male rhesus monkeys (*Macaca mulatta*) participated in these experiments. All procedures were approved by the local authorities (Regierungspräsidium Tübingen) and were in full compliance with the guidelines of the European Community (EUVD 86/609/EEC) for the care and use of laboratory animals. The animals were socially (group-) housed in an enriched environment under daily veterinary supervision. All surgical procedures were performed under aseptic and sterile conditions. Briefly, neural responses were recorded using a custom-made multi-electrode system from alert animals that were passively listening to acoustic stimuli in a dark and anechoic booth. Neural signals were amplified using an Alpha Omega system (Alpha Omega GmbH), filtered between 4 Hz and 9 kHz and digitized at 20.83 kHz. The general procedures used in this study have been previously published (Dahl et al., 2009; Remedios et al., 2009, 2010). To approach the claustrum recording chambers were positioned based on pre-operative magnetic resonance (MR) images and stereotaxic coordinates. In one animal the claustrum was targeted at an angle of 20° anterio-posterior (AP) and 45° dorso-ventrally (DV) so that recordings were centered about at AP +14 mm, DV +18 mm. In the other animal the claustrum was approached vertically, with recordings centered about at AP +18 mm, DV +17 mm. Details of claustral approach and assignment of recording sites to this structure can be found in previous work (Remedios et al., 2010). In general, we sampled many sites along multiple penetrations through the claustrum but here analyzed only those responding to acoustic stimuli. In addition, the neurons analyzed here were all recorded in the “auditory zone”, located roughly half-way between the dorsal and ventral ends of the claustrum (see Figure 1A). However, it should be noted that this zone has been defined purely based on functional properties of the recorded neurons and not based on detailed histological maps. Hence, the recording locations in Figure 1A are only approximate; see (Remedios et al., 2010) for additional discussion.

In the present study we analyzed data from two experimental paradigms involving different acoustic stimuli. The first paradigm has been used previously to study the sound category preferences of neurons at different stages of auditory pathways (Remedios et al., 2009; Perrodin et al., 2011). This stimulus set consisted of 15 sounds each in three categories: (1) macaque vocalizations (Mvoc); (2) vocalizations and noises of other animals (Avoc); and (3) environmental sounds (Esnd) (45 different sounds in total). The Mvoc comprised five call types (coos, grunts, barks, pant-threats and screams), sounds of other animals ranged from birds to lions, horses and tigers and Esnd including noises such as produced by wind, water, doors or jungle background sound. All sounds were sampled at 22.1 kHz and lasted between 0.35 and 1 s and had an intensity of 65 dB root mean square (rms) value. These sounds were presented as a pseudo-random sequence with silent gaps of 1 s in between, and each sound was repeated at least twice. The second paradigm consisted of brief target sounds presented on a background of pink noise (65 dB rms). A pink noise background was used with a similar overall spectrum as the set of sounds used in the first paradigm. The target sounds were a brief white noise burst (80 ms duration), a naturalistic sound (monkey vocalization, grunt, a contextual call that facilitates non-aggressive encounters, 80 ms) or a 300 ms long white noise burst. As control condition, trials without target sound and only presenting the background were included (baseline condition). These targets were presented 500 ms after the onset of the background and were presented at three relative intensities (measured as rms) relative to the background (+0, +6, +12 dB). For each recording site each of these conditions (target types × intensities, baseline) was repeated at least 8 times. We do not report results for the 300 ms noise target, as they were qualitatively and quantitatively very comparable to those obtained for the 80 ms noise target. All sounds were presented from two calibrated free field speakers (JBL Professional) positioned 70 cm from the head and 50° to left and right.

The data was analyzed in Matlab (MathWorks Inc.). Spike-sorted activity was extracted using commercial spike-sorting software (Plexon Offline Sorter, Plexon Inc.) after high-pass filtering the raw signal at 500 Hz. For the present analysis we did not distinguish between single and multi-unit sites. The use of multi-units may in principle influence some of the results on sound selectivity; e.g., individual neurons could be more acoustically selective than derived from our analysis. However, this seems unlikely given that individual responses were very transient, leaving little room for differential selectivity that would disappear in an aggregate response. In addition, the use of multi-unit responses would not affect the interpretation of the sound detection analysis, as mechanistically such a function would most likely be embodied by multiple neurons.

**Figure 2:**
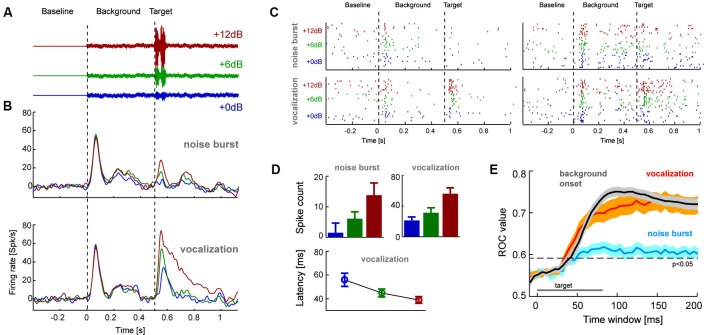
**(A)** The second experiment presented target sounds embedded in a pink noise background sound. Targets had three relative signal to noise ratios (SNR) to the background and were either a short (80 ms) white noise burst or naturalistic sound (conspecific vocalization, 80 ms; shown here). **(B)** Population averaged response time course for each target type and SNR (*n* = 53 units; left). **(C)** Example single trial rasters from two units. Each tick denotes one action potential and different lines show different repeats of the same stimulus condition. **(D)** Average response amplitudes for the target sounds computed in 80 ms windows (shifted by the overall mean latency; 45 ms), and response latency for vocalization targets. Error-bars denote mean and s.e.m. **(E)** Results from a single trial sound detection analysis quantified by receiver operator characteristic (ROC) scores (area under the ROC curve). Lines denote the mean and shaded areas the s.e.m. across units for each target type and the onset of the background sound. Time is indicated relative to the event-onset (either background or target sound) and the ROC scores are based on the cumulative response amplitude in the respective window duration. The indicated significance level (relative to chance performance) was obtained from a randomization test.

Significant responses were determined using a threshold of three standard deviations of the variability of baseline activity. Only those units were included for group analysis that responded significantly to at least one acoustic condition (Mvoc, Avoc or Esnd in paradigm 1; or that responded to the onset of the background noise in paradigm 2). This resulted in the inclusion of 45 units for paradigm 1 and 53 for paradigm 2 (out of 128 unit sampled with paradigm 1; 120 with paradigm 2). For further analysis the responses of each unit to each stimulus were adjusted for differences in spontaneous firing rate by subtracting the average spike count in pre-stimulus period (−400 to −50 ms). For paradigm 1 response amplitudes were calculated for each sound in a 100 ms window centered on the peak of the response time course to this specific sound (with the peak constrained to be within the stimulation period). Amplitudes were then averaged across sounds within each category. The preferred category was defined as that yielding the strongest response amplitude, as in previous work (Remedios et al., 2009). Using shorter windows (e.g., 75 ms) did not qualitatively affect the results. For paradigm 2 the response amplitude for target sounds (Figure 2D) was calculated in 80 ms time windows and with an average latency of 46 ms relative to target onset (derived from a separate latency analysis). Response latencies to target sounds (Figure 2D) were calculated as the first time point of the trial-averaged response crossing the 95% percentile of the distribution of response values in a (300 ms) pre-event period. For this analysis responses were smoothed with a Gaussian window of 10 ms (half-width at half-height).

The analysis for single-trial stimulus detection proceeded as follows. One analysis compared the response amplitudes during target presentation (pooling all signal to noise ratios (SNRs)) to those during a baseline condition provided by trials in which no target was presented. A second analysis compared the response amplitudes following background onset to a pre-stimulus baseline. The receiver operator characteristic (ROC) was calculated by applying a variable threshold to both sets of response amplitudes (targets, baseline) and computing the true and false positive rates for detecting a stimulus. The area under the ROC curve was then used as index to compare detection performance across target types. This analysis was performed based on responses in windows of progressively longer duration (starting at 10 ms pre-event and lasting up to 200 ms post-event onset). To test the statistical significance of ROC values against the null hypothesis of no systematic performance we performed a randomization test in which baseline and stimulus conditions were shuffled (500 permutations). Other statistical comparisons were mostly based on two-way ANOVAs or paired *t*-tests.

## Results

In previous work we have employed an audio-visual paradigm and identified two spatially separated populations of neurons within the claustrum: an auditory zone in which neurons predominantly respond to acoustic stimuli and a visual zone where neurons predominantly respond to visual stimuli (Figure 1A; Remedios et al., 2010). We here explore the response properties of the acoustically responsive zone in more detail. In the following we report findings from two experiments, one testing the general sound category selectivity and one more specifically testing the hypothesis that claustral neurons may function to report the onset of newly appearing sounds.

### Claustral neurons lack selectivity to sound category

We first presented stimuli pertaining to three natural sound categories (Mvoc: conspecific vocalizations, Avoc: sounds of other animals, Ensd: environmental sounds) that have been used in previous studies to establish selectivity of neurons in anterior auditory regions (Perrodin et al., 2011), the posterior insula and caudal auditory cortex (Remedios et al., 2009). Figure 1B displays the responses of all responsive claustral units (both single- and multi-units were included in the same analysis) to the three sound categories and illustrates the general response features in this paradigm. As with most brain regions we expected the claustrum to be a heterogeneous structure comprising neurons with varying selectivity and response properties. The data show that most units exhibited a strong transient onset response within the first 200 ms, which was followed by a more transient response only for a few of the units (Figure 1B). Also, only a few units responded with longer latencies (>100 ms) during the stimulus period, but not at the immediate sound onset. Figure 1C shows the responses of four examples in more detail. Of these, all show a transient response to sound onset and three exhibited very comparable responses across sound categories, while one responded more strongly to the conspecific vocalizations.

This overall insensitivity to sound category was generally true for the entire population. To quantify the selectivity of each unit to the three sound categories we first calculated each unit’s peak response to each individual sound and then averaged response amplitudes within each category. Using individual windows to quantify responses to different sounds ensures that this analysis is insensitive to differences in response latency or time course and to differences in sound duration across stimuli. This result is shown in Figure 1D as scatter plots, which indicate each unit’s average response to the different categories and the variability within each category (standard error across sounds). In general, there was considerably variability of response amplitude across sounds within each category, and the average coefficient of variation (standard deviation divided by mean) was 0.88 ± 0.04 (mean ± s.e.m.). Nevertheless, across units the data scatter along the diagonal, hence revealing differences in the overall response amplitude between units but no systematic effect of sound category. This is further substantiated in Figure 1E, which displays the population average responses (21 ± 3 Spk/s for Mvoc, 17 ± 3 for Avoc, 15 ± 4 for Esnd; mean ± s.e.m.). Statistical assessment revealed no effect of category (ANOVA *F*_(3,179)_ = 0.8, *p* = 0.4). In addition, the fraction of units preferring each of the three sound categories was very comparable (Figure 1E; chi-square test χ = 0.9, *p* = 0.13).

In sum, we found that claustral neurons exhibit strong transient responses to the onset of natural sounds, but as a population do not show a specific preference for any of the tested sound categories. This lack of selectivity differs from data obtained in the insula (Remedios et al., 2009) and anterior auditory regions (Perrodin et al., 2011), where a clear and significant preference for conspecific vocalizations was observed; it also differs from data obtained in primary auditory fields where responses to conspecific vocalizations were weaker than those for the other sounds (Remedios et al., 2009).

### Claustral neurons detect changes in the auditory scene and salient events

These neurons hence exhibit two properties that seem to argue against a primary function in representing acoustic features or sound identity. First, the responses show a lack of selectivity for a specific sound category, and second, responses are very transient even for sounds lasting several hundreds of milliseconds. This suggests that these neurons are more sensitive to the generic onset of new sounds rather than acoustic qualities. This prompted us to test the hypothesis that the claustrum may potentially function as a detector of newly occurring sounds within an auditory scene.

In a second experiment we recorded additional claustral neurons (*n* = 53 responsive units) in response to a paradigm involving the appearance of a target sound amidst continuous background noise (Figure 2). The target was either a short (80 ms duration) white noise burst or a naturalistic sound (monkey vocalization; 80 ms). We chose a vocalization because of its ethological and behavioral relevance. These targets were presented on a pink noise background at various relative SNR (+0, +6 or +12 dB, see Figure 2A). Analysis of response time courses showed that claustral neurons responded well to the onset of the background noise (at *t* = 0 s) and responded with variable amplitudes to the different targets (at *t* = 0.5 s; Figure 2B). Figure 2C furthermore displays the single trial responses of two example-units in this paradigm. Across units, target evoked responses scaled with SNR (computed in 80 ms windows; Figure 2D): an ANOVA (units and SNRs as factors) showed that the effect of SNR was significant for each target type (noise burst: *F*_(2,158)_ = 7.2, *p* < 0.01; vocalization: *F* = 39, *p* < 10^−10^). In addition, target-evoked responses were overall higher for the vocalization compared to the noise (paired *t*-test *p* < 10^−5^, responses averaged across SNRs). Closer inspection of the responses to the vocalization target also indicated a possible effect of response latency (c.f. Figure 2B). We hence analyzed response latencies in more detail. In the vocalization condition target-evoked response latencies could be obtained (for all SNRs) for 26 of the units. For this subset of units, latencies systematically decreased with SNR (56 ± 6 ms, 44 ± 3 ms and 38 ± 3 respectively; mean ± s.e.m.; Figure 2D) and an ANOVA returned a significant effect of SNR (*F*_(2,77)_ = 6.2, *p* < 0.01). Hence, target-evoked responses scale in amplitude and latency with the relative intensity of the target sound. This suggests that claustral neurons identified sounds in a noisy background and responded with firing rate and latency changes.

We predicted that one should be able to decode changes in the auditory scene above chance using claustral responses. A change in the auditory scene could here either be the onset of the background relative to silence or the onset of the target sound relative to background. Given that target-evoked responses were stronger for the vocalization compared to the noise burst, we predicted that this effect should be stronger for the vocalization. To test these hypotheses we performed a single trial detection analysis based on the spike count in a time window of interest. These windows were either aligned to the onset of the background (quantifying how well responses differentiate this from silence) or to the onset of a target (quantifying how well responses differentiate target onset from background noise). A threshold was applied to these response amplitudes to differentiate the condition of interest from responses sampled either during silence or the background. By varying this threshold we calculated the respective receiver operator characteristic (ROC; see Section Materials and Methods), which indicates how well the claustral neurons could serve to detect the onset of new sounds on a single trial basis. This analysis was performed based on responses in windows of variable length, providing an estimate of effect size and the stimulus period required to achieve above chance performance.

We first calculated the ROC for detecting the onset of the background relative to silence. The respective ROC scores were high (peak value 0.76 ± 0.02; mean ± s.e.m.; reached 100 ms after target onset; Figure 2E gray) and significantly above change level (*p* < 0.01). We then calculated the ROC score for detecting the target sounds from background. Importantly, we performed this analysis by pooling responses across SNR conditions, hence mimicking a condition in which a target sound of arbitrary intensity has to be detected. ROC scores were higher for detecting the vocalization (0.75 ± 0.02; peak at 160 ms) than for the noise burst (0.62 ± 0.02; peak at 110 ms; Figure 2E). When compared at the same time point (chosen at *t* = 125 ms; defined based on 45 ms average latency and 80 ms target duration), ROC values differed significantly between noise and vocalization targets (paired *t*-test; *p* < 10^−5^) and between noise target and background onset (*p* < 10^−8^). However, they did not differ between vocalization target and background onset (*p* = 0.33). This suggests that claustral neurons could serve to detect changes in an auditory scene flexibly across a range of SNR values, and they could do so regardless of the specific nature of the target sound.

## Discussion

Studying neurons in the claustrum’s auditory zone, we found that their response properties point to a role in encoding sound occurrence more than sound category or acoustic qualities. In particular we found that this population of neurons was not sensitive to the overall category of sounds and responded with similar strength and transiently to conspecific vocalizations and other naturalistic sounds. This raises concerns as to a function specifically related to encoding sound type or acoustic detail. However, we found that responses within the same claustral zone could serve well to detect the onset of sounds from silence or the onset of novel sounds within an acoustic background. These results are consistent with previous speculations about a role of the claustrum in novelty or saliency (i.e., critically different from the existing background) (Remedios et al., 2010).

### The claustrum and change detection

Detecting a change or novel event in the external environment is of paramount importance for survival. An animal may have to respond to a sudden threat, such as an aggressor or predator that may have remained camouflaged until attack, or it may have to attend to the calls of its offspring in need of immediate attention. The claustrum may fulfill such an ethological role by virtue of its widespread anatomical connectivity. It could detect sudden changes in the environment across sensory modalities, possibly relying on the detection in any sensory modality or relying on the detection in multiple modalities. The claustrum could then send out a generalized awareness signal across its connectome to recruit cognitive or attentional mechanisms to respond to the environmental change. Our results are well compatible with the claustrum participating in a general novelty or vigilance network.

For most mammals sudden environmental events are best detected based on acoustic cues. Hearing serves as warning sensory modality regardless of fatigue, sleep and regardless of current gaze direction, and saliency networks hence must critically rely on some auditory sensitive structures. The claustrum’s auditory zone may be one such hub in a saliency network. Given claustral projections to structures involved in cognitive and motor control (Pearson et al., 1982; Clascá et al., 1992; Smith and Alloway, 2010; Smith et al., 2012) the claustrum could trigger appropriate behavioral reactions or guide the deployment of additional cognitive resources. Parts of the claustrum are well connected with somatosensory and motor structures and could trigger the rapid and coordinated motor response to a novel sound (Clascá et al., 1992; Smith and Alloway, 2010).

It is important to note that we studied claustral neurons only in the context of acoustic stimuli and our findings hence do not speak about the many other claustral neurons that are sensitive to other modalities (Olson and Graybiel, 1980; Remedios et al., 2010). However, our conclusion is well consistent with findings in the visual zone. Sherk and LeVay recorded neurons in the visual claustrum of the cat and found that these neurons were broadly tuned for orientation, with a trend to large receptive fields concerning the visual periphery, but generally being sensitive to visual motion (LeVay and Sherk, 1981; Sherk and Levay, 1981a,b). These visual neurons are hence ideally suited to detect motion in the visual periphery, the location where predators or other important objects usually appear. It will be interesting for future studies to generally compare the tuning and selectivity of claustral sensory neurons in comparison to their ability to report the simple occurrence of sensory stimuli, in order to quantitatively assess the hypothesis of novelty detector across the different zones of the claustrum.

Additional evidence for a role of the claustrum as novelty detector may be provided by a relation between attentional deficits in Autism spectrum disorders (ASD) and changes in claustral volume in affected individuals. Based on structural imaging it was reported that individuals affected by ASD have a smaller claustral volume compared to control subjects (WB, 2008). The behavioral deficits seen in ASD in turn have been linked to changes in attentional deployment (Klin et al., 2003; Ames and Fletcher-Watson, 2010) and one theory holds that a saliency network prominently involving the insula and possibly neighboring structures is critically involved in ASD (Uddin and Menon, 2009). While not providing direct support for our hypothesis, results such as these are well consistent with the notion of a role of the claustrum in sensory detection.

The hypothesis of a network involved in detecting exogeneous change capitalizes on the claustrum’s widespread connectivity with cortical and subcortical structures. Indeed, this structure is ideally placed in order to facilitate the interaction between limbic, sensory and cognitive systems by means of its diverse connectivity, to mediate exchange of sensory or cognitive information or coordinate large-scale activity under challenging circumstances, such as fight or flight situations or mental insight problem solving (Tian et al., 2011; Remedios, 2012; Smythies et al., 2012). Evidence from claustral lesions clearly pinpoint the severe impact of claustral lesions on behavior. Bilateral lesions by herpes encephalitis or mushroom poisoning (Kimura et al., 1994; Nishizawa, 2005) were found to induce severe encephalopathy with disturbance of consciousness, seizures, and psychotic symptoms. In addition, preliminary evidence from targeted claustral lesions in animal models suggest specific behavioral deficits as seen in ASD and large-scale changes in functional brain connectivity (Remedios, 2012). In this context it will also be interesting to understand the relation of the claustrum to neuromodulatory structures. Stimulus novelty also activates the noradrenergic system (Krebs et al., 2013), and relevant structures such as the locus coeruleus interact with limbic structures to facilitate learning during aversive events (Sears et al., 2013). It may well be that the claustrum plays a central role in the formation of memories that facilitate future reactions based on a joint interaction between the claustrum, neuromodulatory systems and the cortex. Further progress towards an improved understanding of the contribution of the claustrum to brain function and cognition hence may benefit from advanced technologies to specifically manipulate activity within this structure or to selectively activate connections between the claustrum and other brain structures (Deisseroth, 2011).

When interpreting the present data it is important to note that the concepts of stimulus novelty or saliency are often used in a loose manner. In particular, in our experiment we did not manipulate stimulus salience independently of stimulus onset or stimulus intensity. While models of acoustic saliency exist (e.g., Kayser et al., 2005), it is generally the case that the onset of a new sound is often the most salient feature in a complex acoustic scene. Hence, any saliency mechanism would also respond strongly to the onset of new sounds. We therefore believe that our findings are well consistent with a role of the claustrum in a more specialized saliency network. Work on the neural underpinnings of visual saliency reported enhanced responses to salient stimuli in those regions potentially implementing the respective saliency map (Constantinidis and Steinmetz, 2005; Arcizet et al., 2011). Interestingly, the neural responses in these areas also scale with the intensity of visual stimuli across a wide range of intensities and exhibit systematically shorter latencies for more salient stimuli (Tanaka et al., 2013). These response properties directly match those observed in the claustrum, where we found stronger and shorter latency responses to more salient (i.e., higher SNR) sounds embedded on a background. Overall it is hence possible that claustral units specifically encode stimulus saliency, but direct tests remain challenging given the difficulty to disentangle saliency and novelty in complex sensory scenes.

To conclude, the role as change or saliency detector provides a data-driven and working hypothesis for future work. It directly suggests experimental paradigms that could be employed in future work to further elucidate this hypothesis and test it in relation to behavior. For example, the detection of salient events by claustral neurons could be probed directed in relation to behavioral detection or to existing algorithmic models of saliency detection in natural scenes (Itti and Koch, 2001; Kayser et al., 2005). Such studies should also test this hypothesis in relation to other sensory modalities, such as the detection of behaviorally or physically salient visual or somatosensory stimuli. A structure with such a widespread connectivity likely has a rather general and amodal (or multisensory) function rather than one pertaining to detailed sensory representations for individual modalities. Hence, any damage of this structure likely results in general brain dysfunction such as seizures, lack of cognitive focus or general psychotic symptoms, which make the interpretation of behavioral deficits following manipulation of claustral activity even more challenging. It will surely remain a challenge to pinpoint the specific function of the claustrum for many coming years, but hypotheses about its putative function are utterly needed to guide future work.

## Conflict of interest statement

The authors declare that the research was conducted in the absence of any commercial or financial relationships that could be construed as a potential conflict of interest.
